# Mapping drug biology to disease genetics to discover drug impacts on the human phenome

**DOI:** 10.1093/bioadv/vbae038

**Published:** 2024-03-09

**Authors:** Mamoon Habib, Panagiotis Nikolaos Lalagkas, Rachel D Melamed

**Affiliations:** Department of Computer Science, University of Massachusetts Lowell, Lowell, MA 01854, United States; Department of Biological Science, University of Massachusetts Lowell, Lowell, MA 01854, United States; Department of Biological Science, University of Massachusetts Lowell, Lowell, MA 01854, United States

## Abstract

**Motivation:**

Medications can have unexpected effects on disease, including not only harmful drug side effects, but also beneficial drug repurposing. These effects on disease may result from hidden influences of drugs on disease gene networks. Then, discovering how biological effects of drugs relate to disease biology can both provide insight into the mechanism of latent drug effects, and can help predict new effects.

**Results:**

Here, we develop Draphnet, a model that integrates molecular data on 429 drugs and gene associations of nearly 200 common phenotypes to learn a network that explains drug effects on disease in terms of these molecular signals. We present evidence that our method can both predict drug effects, and can provide insight into the biology of unexpected drug effects on disease. Using Draphnet to map a drug’s known molecular effects to downstream effects on the disease genome, we put forward disease genes impacted by drugs, and we suggest a new grouping of drugs based on shared effects on the disease genome. Our approach has multiple applications, including predicting drug uses and learning drug biology, with implications for personalized medicine.

**Availability and implementation:**

Code to reproduce the analysis is available at https://github.com/RDMelamed/drug-phenome

## 1 Introduction

Thousands of drugs are FDA-approved, and some unexpected health benefits and risks have been uncovered only after these drugs come into common use. Notably, some drugs have hidden influence on diseases of major public health importance (Gronich and Rennert 2013, Geifman *et al.* 2017). This suggests opportunities for drug repurposing, or for disease prevention. An increasing number of data sources describe the effects of drugs: SIDER (Kuhn *et al.* 2016), DrugBank (Law *et al.* 2014), and the Drug Repurposing Hub (Corsello *et al.* 2017) compile known drug effects on human disease. Systematic information on drug molecular properties are also cataloged, including drug gene targets, and the EPA ToxCast/Tox21 assays of drug biological effects (Richard *et al.* 2016, Subramanian *et al.* 2017). Therefore, methods that can exploit existing data to understand and predict drug effects could have a significant impact on public health.

A number of methods mine data to predict drug effects. Using genome-wide association studies (GWASs) to link phenotypes to likely causal genes, researchers showed that effective drugs for a disease target disease genes (Nelson *et al.* 2015), and, similarly, that genetics can predict drug side effects (Nguyen *et al.* 2019). A popular method, the connectivity score, proposes that effective drugs for a disease will induce an expression profile that contrasts with the disease expression profile (Dudley *et al.* 2011, Sirota *et al.* 2011, Chen *et al.* 2017). In a variation on this approach, So *et al.* (2017) contrasted drug-induced gene expression with disease-associated gene regulation. Specifically, they used the S-PrediXcan method (Gamazon *et al.* 2015, Barbeira *et al.* 2018), which estimates the association of disease risk with regulation of expression of each gene. Other computational methods for discovering drug effects propose that drugs with more similar molecular effects will have more similar phenotypic effects (Gottlieb *et al.* 2011). The same premise has motivated recent work using matrix completion to find drug effects (Wang *et al.* 2017, Bakal *et al.* 2020, Galeano *et al.* 2020, Lau and So 2020, Zhang *et al.* 2020).

The approaches described above have focused only on predicting drug effects, rather than learning how drug molecular effects relate to disease biology. Here, we aim to learn how drug impact on disease can be explained by the relationship between the biological effects of the drug and the genetic alterations driving disease. Therefore, we represent each drug and disease by a profile of the molecular changes associated with drug (from ToxCast) or disease phenotype (S-PrediXcan). We propose that simple linear models connecting a drug’s molecular effects to a disease’s genetic drivers can explain the drug’s effect on phenotype. To estimate this interaction matrix, we take a supervised approach, training the model based on known drug impacts on disease. Importantly, by simultaneously training this model to predict relationships between tens of thousands of drug–disease pairs in a multitask fashion, we aim to learn an interpretable network connecting drugs to phenotypes. We call this method Draphnet, or Drug and Phenotype Network.

Learning this network has a number of advantages. First, interpretability provides a rationale for predictions, increasing confidence in these predictions. Second, this model can provide testable hypotheses for future analysis of drug-disease biology. Third, these findings can provide new insight into the biological basis of known drug phenotypic effects, which are often poorly understood. This can allow a new classification of drugs based on their downstream effects on disease biology. We describe: (i) our development and implementation of Draphnet, (ii) evidence supporting Draphnet’s ability to predict drug–phenotype relationships and recapitulate known drug biology, and (iii) Draphnet’s application for gaining new insight into drug biology.

## 2 Methods

### 2.1 Preparation of the disease genetic gene expression profiles and linking to drug phenotype data

GWAS results for many UK Biobank phenotypes have been made publicly available (Rapid GWAS of Thousands of Phenotypes for 337,000 Samples in the UK Biobank). For each GWAS, the PhenomeXcan resource compiles gene-based associations for dozens of human tissues using S-PrediXcan, and combines these results across tissues using the S-MultiXcan method to create a *P*-value for each gene and phenotype combination (Pividori *et al.* 2020). We follow the method in PhenomeXcan to convert these statistics into a *z*-score that represents strength of the association of the phenotype with variants that increase (positive *z*-score) or decrease (negative *z*-score) gene expression. First, we convert each S-MultiXcan *P*-value (${\mathrm{multiXcanPvalue}}_{\mathrm{gene},\mathrm{phenotype}}$) associating a gene with a phenotype to a *z*-score statistic using the inverse normal cumulative distribution, $\Phi^{-1}({\mathrm{multiXcanPvalue}}_{\mathrm{gene},\mathrm{phenotype}})$, where Φ represents the standard normal distribution function. Then, we obtain the consensus (majority) S-PrediXcan sign (positive or negative regulation) for a gene across all tissues, ${\mathrm{sign}}_{\mathrm{gene},\mathrm{phenotype}}$. Therefore, our final gene-based score for each phenotype is ${|\Phi }^{-1}({\mathrm{multiXcanPvalue}}_{\mathrm{gene},\mathrm{phenotype}})|\times {\mathrm{sign}}_{\mathrm{gene},\mathrm{phenotype}}$, or the absolute value of the statistic, times the sign. For simplicity, we refer to these estimates of gene–disease association as PhenomeXcan results. This creates the preliminary matrix *P* with rows as each gene, and columns comprising hundreds of GWAS phenotypes.

To match the GWAS phenotypes in *P* to the SIDER phenotypes in *Y*, we use the Unified Medical Language System (UMLS) to match phenotype names to UMLS concept unique identifiers (Bodenreider 2004). SIDER includes concept unique identifiers indicating phenotypes associated with each drug, allowing us to match the GWAS phenotypes to drug indication and side effect profiles. Finally, we keep only the top half of genes most variably associated with phenotype (highest standard deviation). This design decision keeps genes that are expected to be most informative of phenotype biology, reducing the number of parameters in the model.

### 2.2 Preprocessing of ToxCast data

We obtain the ToxCast data from https://www.epa.gov/chemical-research/exploring-toxcast-data. Each assay tests the effect of multiple concentrations of a compound against some readout of biological effect, known as an endpoint. For each such endpoint, a series of normalization, postprocessing, and modeling steps have already been performed as part of the resource. These are publicly available as Level 5 data, which estimates the fraction of models that call a compound as a “hit” for a particular endpoint. We obtain the public Level 5 data which comprises our matrix *D*. This matrix contains 1391 endpoints for more than 429 compounds, but it contains many missing entries where a compound was not tested for an endpoint.

The affinity regression method learns a bilinear model, and the parameter space can be reduced with dimensionality reduction (see section “Implementation of affinity regression for binary outcomes”). To perform dimensionality reduction of this data, we use the SoftImpute package, which is designed for matrix decomposition in the presence of missing data (Hastie *et al.* 2014). This method finds a singular value decomposition of a matrix $D=U_{D}S_{D}V_{D}^{t}$ that can impute the missing values in the matrix *D*. We emphasize that our goal is not imputation of *D*, but to obtain the best reduced-rank representation of the endpoint data. SoftImpute requires the user to specify the rank of the decomposition, as well as a regularization parameter lambda. To choose these values, we perform a cross-validation-like approach, setting 5% of the nonmissing values to be missing, and quantifying the mean squared error of imputation of these values. After picking these hyperparameters (rank of 246, regularization parameter lambda at 0.34), we project each drug onto this lower dimensional space using the product $U_{D}S_{D}$.

### 2.3 Assessing similarity between drug–phenotype relationships and molecular profiles

To establish the premise of our approach, we assess whether pairs of drugs with more similar molecular profiles also have more similar phenome-wide associations. For each pair of drugs, we calculate the Jaccard index between the two drugs’ binary profiles denoting presence or absence of association with each disease. Then, to quantify the endpoint similarity of a pair of drugs, we calculate the Spearman correlation of their ToxCast endpoint scores, when considering only the endpoints in which both drugs were evaluated. Finally, we calculate the association between Jaccard index and endpoint correlation across drug pairs, using the Spearman correlation coefficient, as well as a linear model modeling side effect similarity as a function of endpoint correlation, accounting for the number of endpoints a pair has in common. We also performed permutations, shuffling the order of the ToxCast drug pair data to assess how often the shared signal between these two data sources would be seen by chance.

Similarly, we estimate whether diseases that are impacted by similar drugs have a similar molecular profile. We perform the analogous calculation: for each pair of phenotypes, we calculate how overlapping their sets of drugs are using the Jaccard index, and we compare this quantity to the correlation of their PhenomeXcan gene associations.

### 2.4 Implementation of affinity regression for binary outcomes

Our model adapts affinity regression (Pelossof *et al.* 2015). In this method, the bilinear regression problem $DWP^{t}=Y$ is transformed to a standard regression by taking the Kronecker product of the feature matrices, denoted ⊗, as follows: (P⊗D)×stack(W)=Y, where stack refers to flattening the coefficient matrix *W* into a vector of coefficients. In this way, the matrix *W* can be learned using a standard regularized regression. Because in our case *Y* is not continuous but binary, we instead used regularized logistic regression to learn the model $DWP^{t}=\mathrm{logit}(p(Y))$. As outlined in Pelossof, *et al.* (Pelossof *et al.* 2015), the large number of parameters in *W* can be reduced by performing a dimensionality reduction on the matrices *D* and *P*. Analogous that paper, we reformulate the regression using matrix decomposition as:


(1) $$DWP^{t}=\mathrm{logit}(p(Y))=(U_{D}S_{D}V_{D}^{t})W(U_{P}S_{P}V_{P}^{t})=(U_{D}S_{D})W_{DP}(V_{P}^{t})$$

where $W_{DP}=\left(V_{D}^{t}\right)W\left(U_{P}S_{P}\right).$

Therefore, we train the model: $\left(V_{P}⊗\left(U_{D}S_{D}\right)\right)\times \mathrm{stack}\left(W_{DP}\right)=\mathrm{logit}\left(p\left(Y\right)\right)$. Our training uses standard lasso logistic regression from the scikit-learn package.

As described above, we represent each drug using the lower rank matrix $U_{D}S_{D}$ learned using SoftImpute. We decompose *P* as well, which can be done using standard singular value decomposition (SVD): $P^{t}=U_{P}S_{P}V_{P}^{t}$.

To train the model, we then tune hyperparameters by holding out data on 10% of drugs in each fold of the cross validation to find the parameters that result in the best prediction accuracy on held out drugs. The hyperparameters are the regularization parameter for the logistic regression, as well as the truncated ranks of the SoftImpute and SVD decompositions, $r_{P}$ and $r_{D}$. For the indications model, our final hyperparameters are $r_{P}$ = 131, and $r_{D}$ = 95 and the best regularization parameter for the lasso logistic regression was 1. For the side effects model, our hyperparameters were $r_{P}$ = 197, and $r_{D}$ = 246 and lasso regularization parameter 0.1.

### 2.5 Evaluation of predicted drug side effects

To evaluate the predictive performance of our model, we approximate a leave-one-drug-out model by performing a 20-fold cross validation analysis. A leave-one-drug-out model would be expected to perform even better as it would be trained with more data. For each fold, we obtain hyperparameters and predictions of drug side effects for the held-out 5% of drugs. To compare to a baseline model, we obtain predictions for each drug by using the drug’s nearest neighbor in the ToxCast data as a predictor of that drug’s side effects. We compare these two types of predictions in Fig. 2A.

We also compare our side effect predictions to those of Galeano’s recommender model (Galeano *et al.* 2020) and Oh’s Drug Voyager (Oh *et al.* 2017), with both of SIDER and Offsides (Tatonetti *et al.* 2012) as comparison points for the models. For this purpose, drugs were mapped to DrugBank and PubChem identifiers, and phenotypes were mapped to UMLS or MedDra terms.

### 2.6 Mapping drugs to their phenome and disease genome effects

In order to map each drug onto the space of its effects on diseases, we multiply the transformed drug endpoint data $U_{D}S_{D}$ with the learned lower-dimensional matrix $W_{DP}$. We call this product $U_{D}S_{D}W_{DP}$ the “drug phenome matrix” because it maps each drug to the space $r_{P}$ representing a compressed summary of the effects of drugs on the phenome (see Table 1).

**Table 1.** Summary of matrices.

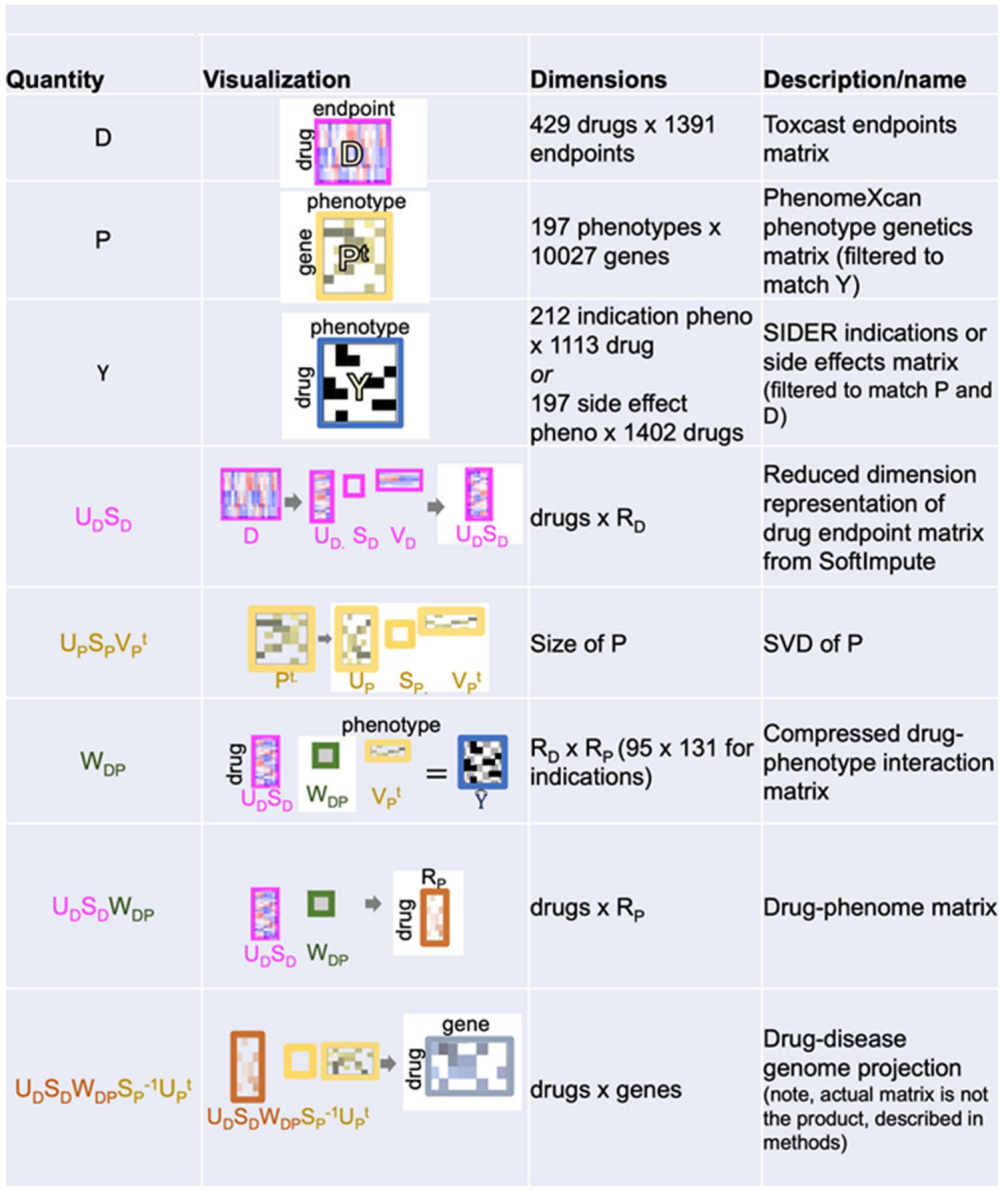

We can further decompress this representation to reconstruct the higher dimensional “drug disease genome matrix.” Using the inverses of the matrices from the SVD of *P*, we calculate the product:


(2) $${(U}_{D}S_{D}W_{DP})S_{P}^{-1}U_{P}^{t}$$

Note that $U_{P}^{t}$ is an orthogonal matrix (see Equation 1). As a result, we project each drug onto the space of phenotype genetics (here, 10 027 genes with variation in regulation associated with GWAS phenotypes).

In order to assess the importance of each connection between a drug and a disease gene, we create a null distribution through permutation. Specifically, we permute the values of *Y* and then train the model again. We obtain a null drug disease genome matrix ${\tilde{W}}_{DP}$ for each of 10 000 permutations, and create the null drug disease genome matrix using the procedure in Equation (2). Then, for each entry in the drug disease genome matrix, we test whether the true value is more extreme than the distribution of the corresponding drug–gene pair in the permuted data. Finally, we adjust these empirical permutation-based *P*-values for multiple tests (10 027 genes for each drug). For this, we use the Benjamini-Yekutili (Benjamini and Hochberg 1995, Benjamini and Yekutieli 2001) method, which is appropriate for nonindependent hypotheses. In result, we have a *P*-value for the importance of the connection of each drug to each disease gene. Note that these *P*-values represent the significance of the association between a drug and disease gene that is not just due the input data *D*, as the input data remains the same across all permutations.

### 2.7 Drug target and therapeutic class analysis

We obtain molecular targets for each drug from DrugBank and Therapeutic Targets Database (Law *et al.* 2014, Wang *et al.* 2020).

As described in Results, we ask whether drugs that share targets have: (i) more correlated endpoint effects, and (ii) more similar phenome effect vectors. For ToxCast data *D*, and for the projected phenome effect matrix $U_{D}S_{D}W_{DP}$, we obtain the Spearman correlation of the feature vectors for pairs of drugs that share, or do not share, recorded targets. We compare these correlation distributions using the rank sum test.

To demonstrate that our model learns new information beyond that contained in the input data, we evaluate whether our projected drug phenome matrix is able to distinguish drugs that share targets more effectively than a null model. The null model projects each drug using ${\tilde{W}}_{DP}$, a scrambled interaction matrix. We compare rank sum test results for each drug target in the null drug phenome effect matrix $U_{D}S_{D}{\tilde{W}}_{DP}$ versus in the true matrix (Fig. 2C). To assess whether the projections have increased similarity between drugs that share a target, we use the same null model. Finally, we directly assess correlations of the phenome effect vectors among pairs of drugs sharing a target, comparing correlations in the true phenome effect matrix against vectors projected using ${\tilde{W}}_{DP}$ (Fig. 2D).

The disease genome matrix assesses which disease genes each drug is significantly associated with. We ask for each drug target in DrugBank, and for each disease gene associated with one or more of the drugs targeting that gene, if drugs that share that target are enriched for drugs associated with that disease gene. We quantify this overlap using the hypergeometric test, and the results are adjusted for the number of genes tested for each target using the Benjamini–Hochberg procedure. To assess whether drug targets have more significant gene associations than expected by chance, we permute the assignment of drugs to targets and repeat the procedure. For each true drug target and permuted version of that target, we obtain the *P*-value for the most significant disease gene association (Fig. 3B).

### 2.8 Categorizing drugs by association with disease genome

To make the visualization in Fig. 4, we filter the disease genes to keep those significantly associated with one or more targets, and associated with fewer than 15 total drugs.

To make the drug–drug network, we connect pairs of drugs that share more disease genes than expected by chance. To test whether a given pair of drugs share a significant overlap of disease genes, we create a null distribution by simulation. Specifically, we sample random disease genes for one drug of the pair, where disease genes are sampled in proportion to how many drugs they are significantly associated with. Then, we assess the size of the overlap in this random set, and repeat this 1000 times to obtain a nominal significance level based on comparing the true overlap to this distribution.

Finally, we identify fully connected cliques of drugs from this network. We analyze the drug–drug graph with the networkx package, retrieving all fully connected cliques of drugs from this graph. Finally, we use the igraph package to visualize drugs and genes as a bipartite graph.

## 3 Results

### 3.1. Model summary, data curation, and assessment of predictive signal in data

**Figure 1.**
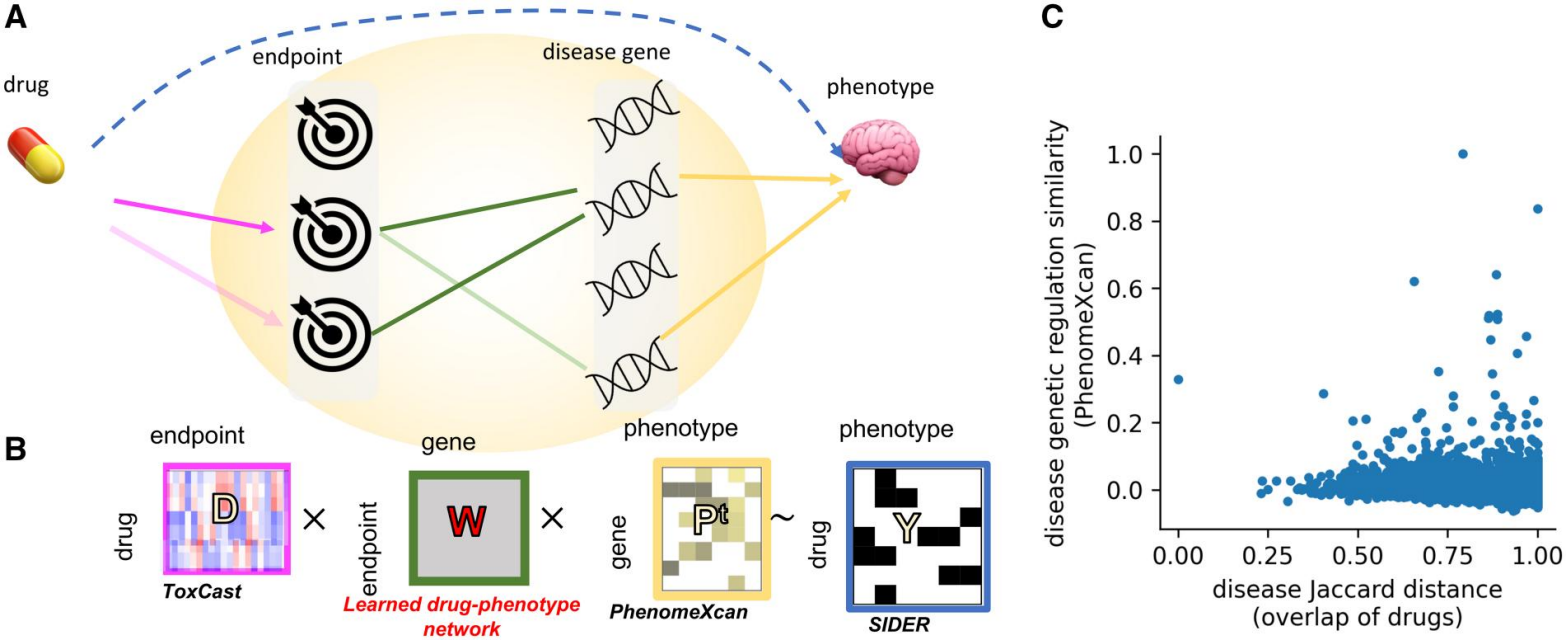
Design and support for the method. (A) Proposed model where a particular drug’s effects on endpoints (pink) can be propagated to impact on disease genes (green), genes in turn are associated with a phenotype (yellow), explaining drug effect on phenotype (dashed blue line). The same endpoint to disease gene network (green) is learned across thousands of pairs of drug and phenotype. (B) Translating the proposed model into an affinity regression integrating: (i) similarity of a drug to all other drugs (ToxCast, *D*), (ii) similarity of a disease to all other diseases (PhenomeXcan, *P^t^*), and (iii) known drug–disease relationships (SIDER, *Y*). Two separate model are learned, one for *Y* as drug side effects, and one for *Y* as drug indications. (C) Each point is one pair of diseases. The *x*-axis shows the Jaccard distance (1 − Jaccard similarity index) and the *y*-axis shows the Spearman correlation of the disease pairs in terms of PhenomeXcan genomic profile. Phenotype pairs with higher PhenomeXcan similarity have lower Jaccard distance (Spearman correlation=−0.11).

We propose that the effects of drugs on key biological processes result in effects on disease genes and proteins, explaining the effect of drug on phenotype (Fig. 1A). Therefore, we expect that by modeling drug phenotypic effect as a function connecting drug biology to genes altered in disease, we can predict and explain drug effects. To implement and test this hypothesis, we aim to learn a model linking the biological processes altered by each drug (summarized in the matrix *D*) to the gene drivers of phenotype risk (in the matrix *P*), where the model is trained to predict the (incomplete) matrix *Y* of drug–phenotype association from SIDER (Fig. 1B). Our design adapts the affinity regression method (Pelossof *et al.* 2015, Osmanbeyoglu *et al.* 2017) developed to model gene regulation. Affinity regression learns the interaction between two sets of input features (here, respective molecular data describing each of drugs and diseases) in order to explain and predict our observed data set (here, the drug–disease associations). In this section we describe the data obtained to build our model and we demonstrate that the molecular data has promising potential for predicting drug–phenotype associations. In subsequent sections we describe evaluation of our model itself for predicting drug–phenotype associations, as well as for gaining insight into drug biology. Our model is trained to predict the SIDER drug–phenotype association matrices *Y*, which are binary: drug–phenotype association are either recorded (1) or not known 0. There are two *Y* matrices, one representing drug side effects and one representing drug indications; therefore, we train two separate models to predict each.

To represent the molecular profile of each drug in the matrix D, we compile EPA ToxCast assays recording a range of 1391 endpoints for hundreds of common medications. For example, one endpoint assay tests the androgen receptor agonist potential of a compound, while another tests the androgen receptor antagonist potential. It is important to note that drugs are not assayed for all endpoints—on an average, each drug is assayed for around 100 endpoints. We evaluate whether similarity among drug ToxCast endpoints is predictive of drug side effects, finding that drugs with more similar ToxCast endpoint profiles were significantly more likely to be associated with the same phenotypes (Spearman correlation = 0.04, *P* = 1e-22, see section “Methods” for details), a signal which is also found when accounting for the number of endpoints assayed per compound (*P* = 1.8e-46, section “Methods”), and which did not appear in permutations of the data (*P* < 1e-3). The affinity regression design can exploit such similarity by training the model on reduced rank feature matrix created by dimensionality reduction. To this end, we use SoftImpute (Hastie *et al.* 2014), a method for dimensionality reduction in the presence of missing data to create a lower dimensional representation of the drug molecular profiles, $U_{D}S_{D}$ (see section “Methods”). Although we doubtless lose some information about each drug’s biological effects, we find a significant correlation between the pairwise similarity of drugs before dimensionality reduction and as projected on $U_{D}S_{D}$ (Spearman correlation = 0.21, *P* < 1e-100 comparing similarity of pairs of drugs from the matrix D vs $U_{D}S_{D}$).

To represent disease biology in the matrix P, we use gene-based results derived from UKBiobank. GWAS of this data has associated loci with risk of thousands of phenotypes (including all common diseases as well as many health traits such as smoking) (Details and Considerations of the UK Biobank GWAS). The PhenomeXcan project integrates these GWAS results with expression quantitative trait locus results linking the same risk loci to expression of each gene (Pividori *et al.* 2020). Therefore, the matrix P contains estimated association of regulation of each gene with presence of disease. Keeping only the genes that vary most highly across phenotypes, we obtain 10 027 genes for 197 phenotypes that can be matched to SIDER phenotypes. Analogous to the evaluation we performed with the drugs, we again ask whether similarity among phenotypes in the space of genome-wide gene regulation is associated with similarity of drug associations. We found a significant relationship (Spearman correlation=−0.11, *P* = 4e-81, Fig. 1C), again supporting the premise that similarity among the input feature vectors in this data set predicts drug–phenotype association. That is, across all available pairs of phenotypes, we show that similarity in genome-wide genetic regulation is associated with a similarity in drug associations; to our knowledge, this signal has never been previously reported.

In summary, we conclude that drugs with more similar molecular profiles are more likely to be associated with the same phenotypes. As well, diseases with more similar PhenomeXcan molecular profiles are also impacted by overlapping sets of drugs. To exploit this signal, we adapt the affinity regression method (Osmanbeyoglu *et al.* 2014, Pelossof *et al.* 2015) to learn a matrix describing how interactions between molecular features manifest in the resulting drug–phenotype associations. While affinity regression has previously been applied to predict continuous (normally distributed) data, here we model a binary outcome (presence of drug–disease relation) in the model: $DWP^{t}=\mathrm{logit}(p(Y))$ (Fig. 1B, see section “Methods”). In effect, we are learning the weighted bipartite network W connecting each drug molecular endpoint to each disease genetic driver. Although the matrix W has many parameters, we impose sparsity constraints, and we additionally reduce the model size by factorizing both D and Pt to lower dimensional representations, as described above for D (section “Methods”). Therefore, we instead learn the smaller matrix $W_{DP}$. Although the biology of drug side effects is expected to be related to that of indications (Wang *et al.* 2014), we train Draphnet separately to predict either drug side effects or drug indications, as these are recorded in separate Y matrices. Matrices are summarized in Table 1.

### 3.2 Assessment of the model’s predictive performance for drug side effects

**Figure 2.**
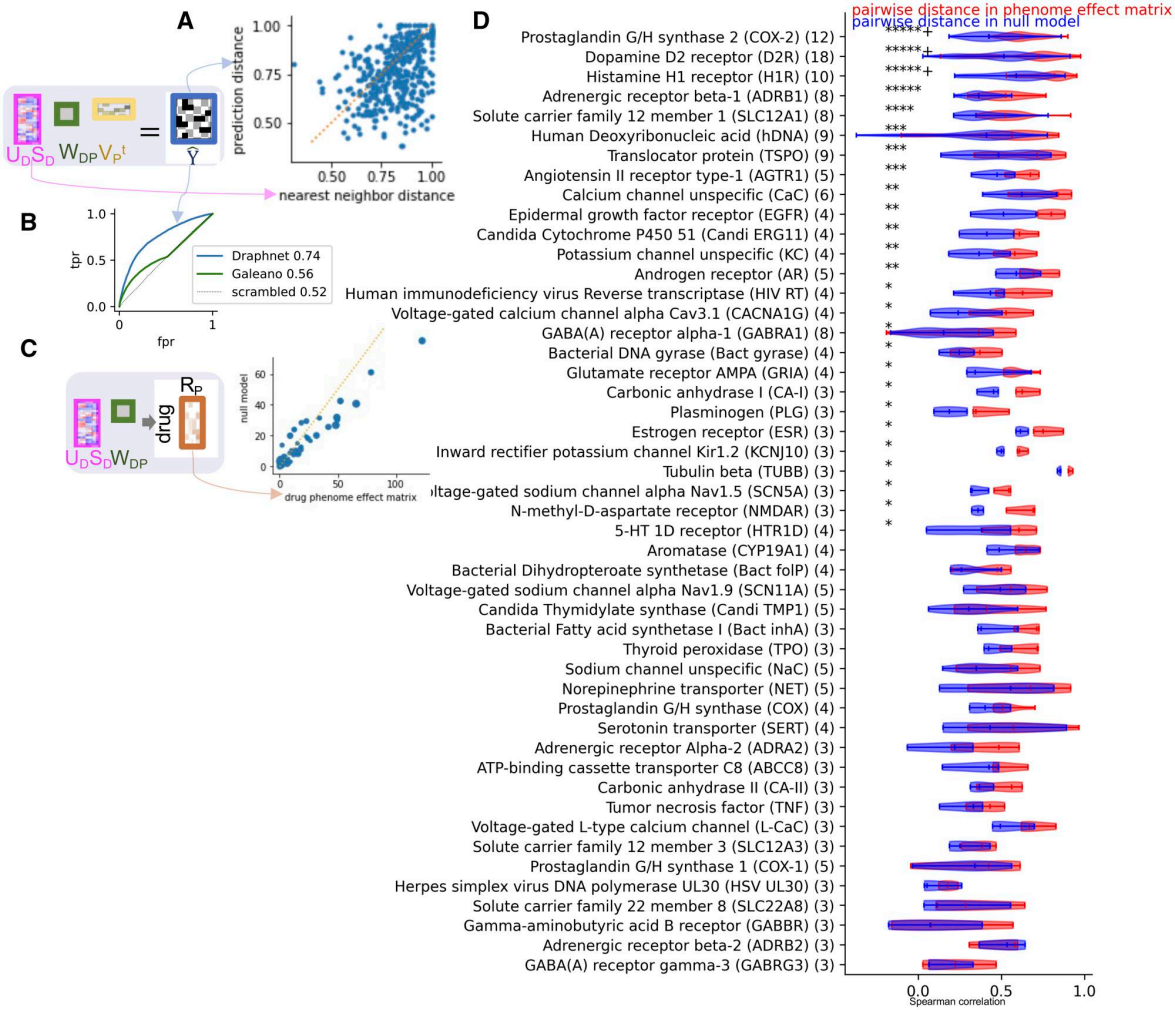
(A) Prediction performance (Jaccard distance between predicted vs held-out drug effects) is substantially improved as compared to baseline predictive model based on side effects of the nearest neighbor (in *U_D_S_D_*). (B) ROC curves and AUC for Draphnet, Galeano’s recommender system, and Draphnet’s drug input data alone, for prediction of SIDER side effects. (C) Each point is one drug target, both axes compare similarity of drugs that share that target versus the similarity of those drugs to other drugs (−log10 *P*-value of rank sum test). (D) Distributions of pairwise Spearman correlation of projections of drugs sharing various targets in projection versus in null projection. Significance is indicated by the stars (“*****+” indicates *P*-value** **<** **10^**−**^^5^). Number of drugs per target is in parentheses.

We first evaluate the model’s generalizability and robustness on tasks it was trained on. Therefore, as an initial assessment of Draphnet, we test its ability to predict drug side effects. First, we ask whether the predictive performance can be explained by the input data alone, or if the model outperforms its input data. Predicting the drug side effects for held out drugs, we find that for a majority of drugs, our predictive model outperforms a nearest neighbor model as baseline (lower Jaccard distance between the predictions and the actual side effect profile, as compared to the nearest neighbor method, *P* = 9e-11, rank sum test) (Fig. 2A). Although very similar drugs in the ToxCast matrix might have very similar side effect profiles, this test shows that the predictive performance cannot be attributed only to information in the input data. This result also shows that Draphnet’s phenotype predictions can generalize to new drugs.

To further evaluate the predictions of drug side effects, we compared Draphnet against other approaches that predict drug side effects. A recommender system created by Galeano *et al.* (2020) was designed to not only predict drug side effects but also predict their frequency. This approach shares some commonalities with Draphnet as both methods can be seen as matrix factorization approaches; as well both approaches were trained using the SIDER data. We evaluate the predictions of presence of a drug–phenotype association on the 215 drugs and 155 phenotypes present in all of ToxCast, SIDER, and the tables available from Galeano *et al.* First, we find moderate correlation between Draphnet’s predicted probability of drug side effect and Galeano’s predicted side effect frequency (Spearman correlation = 0.24, *P* < 10e-300). Then, we assess how well each score predicts presence of SIDER side effect: the predictions from Galeano have an AUC of 0.56, and Draphnet has an AUC of 0.74, and a scrambled version of Draphnet’s interaction matrix has an AUC of 0.52 (Fig. 2B).

Galeano *et al.* also evaluate their predictions as compared to the Offsides database, which measures whether drugs and side effects are disproportionately co-reported in the FDA adverse event reporting system (FAERS) database, as compared to their marginal frequencies (proportional reporting ratio, a continuous value). For the 193 drugs and 155 phenotypes present in Offsides, ToxCast, and Galeano, we found that both Draphnet’s predictions (probability of drug side effect) and Galeano’s scores were significantly correlated with Offsides proportional reporting ratio (Galeano correlation = 0.12, *P* = 8e-105; for Draphnet, 0.14, *P* = 7e-131).

We also compare Draphnet against Drug Voyager, another method that uses molecular data in its predictions (Oh *et al.* 2017). Only 20 drugs and 151 phenotypes were present in ToxCast, SIDER, and Drug Voyager results; the AUC was 0.54 for Drug Voyager and 0.75 for Draphnet. To summarize, we evaluate our model both by performance on held-out SIDER data and on external FAERS data, showing that Draphnet outperforms both its input data and two other approaches with similar goals of predicting drug phenotype effects.

Some interesting drug–phenotype pairs not present in SIDER are ranked highly. For example, among drugs not known to treat eczema, fludrocortisone is most strongly predicted to treat eczema. This drug is an oral corticosteroid, while eczema is typically treated by topical steroids. The highest ranked nonindicated drug for glaucoma is methyclothiazide, a diuretic. As glaucoma’s main cause is fluid retention in the eye, this indication is plausible. In another promising example, dasatinib’s primary indication (leukemias) was not available in our training matrix P, but despite this, the top predicted indications were cancers. Therefore, rather than false positives, these may be true drug effects.

### 3.3 Using the model to map drugs to their effect on the phenome

Although predicting drug effect on phenotype is of interest, the advantage of our approach is its potential to provide insight into the biology of drug effects. To this end, we use our learned interaction matrix to map drug endpoints to their effects on the phenome. The $U_{D}S_{D}$ matrix summarizes the variation in drug endpoints induced by each drug. By multiplying this matrix with the learned matrix $W_{DP}$ we obtain $U_{D}S_{D}W_{DP}$, which maps each drug to a compressed summary of its effect across all phenotypes. Therefore, we call this matrix the drug phenome effect matrix. Next, we investigate whether the drug phenome effect matrix reflects known characteristics of drugs.

First, we confirm that drugs that share one or more known gene targets have more similar ToxCast endpoint effects (*P* = 1e-28, rank sum test comparing similarity of pairs of drugs that share targets against those that do not). To show that the drug phenome effect matrix reflects learned information beyond that captured in the ToxCast matrix D (and, therefore, captured in $U_{D}S_{D}$), we create a null model for the phenome effect matrix. Our null model is obtained by projecting $U_{D}S_{D}$ onto a permuted summary of its effects on the phenome ${{\tilde{W}}_{DP}}_{,}$, by multiplying $U_{D}S_{D}{{\tilde{W}}_{DP}}_{.}$. It is important to note that the input data $U_{D}S_{D}$ is not permuted, so any difference between the performance of $U_{D}S_{D}W_{DP}$ versus $U_{D}S_{D}{{\tilde{W}}_{DP}}_{.}$must be due to interactions of features learned by Draphnet. Comparing the drug phenome effect matrix to this null model allows us to identify the effect of learning the interaction matrix. As this permutation does not nullify the information captured in the endpoint data $U_{D}S_{D}$, in the null projection $U_{D}S_{D}{{\tilde{W}}_{DP}}_{\;}$drugs that share targets are also more similar than ones that do not. However, for most drug targets, the true projection improves our ability to distinguish drugs sharing targets from those that do not, as compared to the null matrix (Fig. 2C).

While some drug target classes do not follow this pattern, this may be due to polypharmacology, which describes the complex nature of the biological effects of drugs. Most of these targets are rather broad. For example, DrugBank annotates 14 drugs as targeting *CHRM1*, and this list include anticholinergics, neuroleptics, migraine treatments, and ophthalmological preparations. These 14 drugs have a median of 19 other targets. This underlines the need for systematic approaches to better understand the biological effects of drugs.

Therefore, we investigate the similarity of drugs of the phenome effect vectors within individual classes of targets (annotated by Therapeutic Targets Database). Comparing pairs of drugs that share targets, their phenome effect similarity is systematically higher in the true drug phenome effect matrix as compared to the same pairs of drugs in the null projection (Fig. 2D). This implies that the learned interaction matrix allows us to learn a representation of drugs that is consistent across drugs sharing known mechanisms of effect, and that this is not just due to the similarity of the drugs in the input data.

### 3.4 Mapping drugs to their effect on the disease genome

To investigate biological insight that can be gained from these mappings, we further project the drug phenome effect matrix to the space of disease genetic regulation, using the inverses of our matrix decomposition (Methods). This projection maps each drug onto the space of its estimated impact on genetic regulation driving disease. Again, we wish to distinguish drug effect on the phenome learned in our model, as opposed to patterns that simply exist in the input data. Therefore, we again compare each drug–disease gene score against the corresponding drug–gene values observed in empirical null models (see Method). As a result, we create a matrix estimating for each drug the importance of its effect on each disease gene, which we call the drug–disease genome matrix (Supplementary Table S1). Because we compare the strength of each drug–gene connection to projections using the same input data (*D* and *P*) but with null models ${\tilde{W}}_{\mathrm{DP}}$, these connections cannot be due only to the prior data on drug molecular effects: the drug–gene relations must be due to the learned interaction matrix that estimates how molecular effects propagate to impact disease (as outlined in Fig. 1). In principle, we can estimate the chance a drug affects a particular disease by taking the dot product of the drug’s disease genome vector with the disease’s PhenomeXcan profile:


$$\sum_{g}\;d\mathrm{rugGeneEffec}t_{g}\times \mathrm{gene}D\mathrm{iseaseEffec}t_{g}.$$

**Figure 3.**
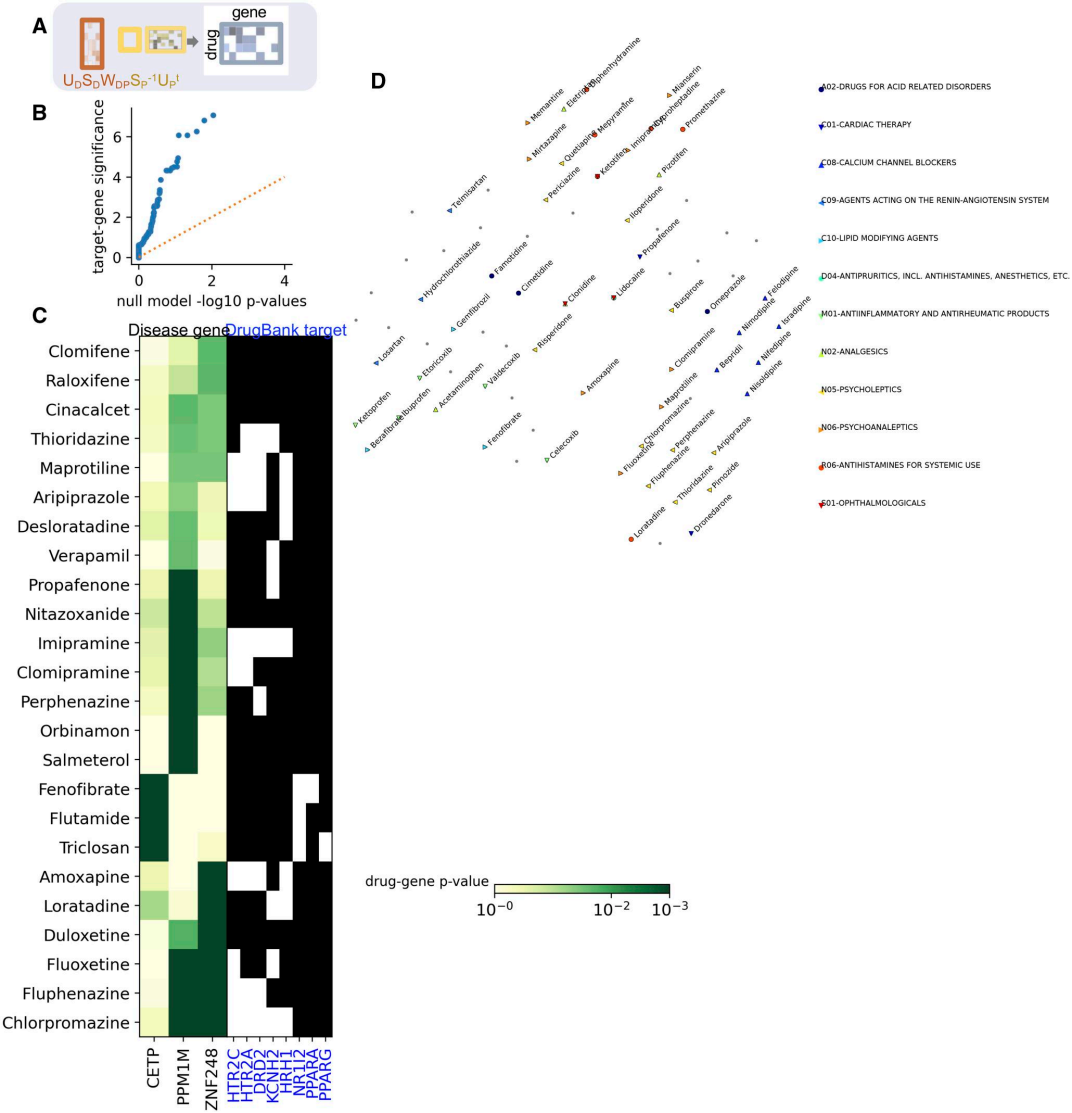
(A) Schematic showing projection of drugs to disease genome. (B) Empirical −log10 *P*-value calibration plot showing distribution of significance for drug target groups versus permuted drug targets. (C) Drug significance for selected drugs with targets significantly associated with the disease genome (green scale showing significance). To the right, selected DrugBank targets of these drugs are shown. (D) tSNE visualization showing similarity of drugs according to their target significance, with ATC therapeutic groups highlighted.

We obtain a median of 7 disease genes associated with each drug. We then assess for each drug target, whether drugs that share that target also share disease genes (see section “Methods”). Across 132 DrugBank targets shared by at least three drugs, we find 28 targets that have one or more significantly associated disease genes at adjusted *P* < .01 (Methods, Supplementary Table S2). Figure 3A shows that this level of association of drug disease genetics and drug targets is not likely to happen by chance. Therefore, we conclude that drugs sharing targets are more likely to impact the same disease genes, supporting the biological relevance of the drug–disease genome matrix.

We visualize the variation in drug–gene associations across drugs in these target groups in Fig. 3B, where each drug is labeled by its ATC therapeutic subgroup. This visualization shows that drugs in the same therapeutic category have more similar gene associations: calcium channel blockers cluster together in one area, and anti-inflammatories and analgesics are in another cluster. We investigate some of the drug–disease gene relationships in Fig. 3C. For example, *PPM1M* is predicted to be impacted by a number of neuroleptic drugs that target *HTR2C*, involved in serotonin signaling. It is plausible that *PPM1M* could be a key driver of the effect of these drugs: it is the top PhenomeXcan gene for bipolar disorder; a recent study found loci in this gene to be associated with schizophrenia (Goes *et al.* 2015); and another study linked the locus to rare mental illness (Mallard *et al.* 2022). Another interesting finding was the association of disease driver *CETP*, or cholesterol ester transfer protein, with fenofibrate and other drugs targeting lipid metabolism. This gene is associated with high cholesterol in the PhenomeXcan results (though not one of the top associated genes). Supporting a true effect of drugs on this driver, this gene has been associated with the effects of fenofibrate and *PPARɑ* agonism in experimental work (Hansen *et al.* 2010, Armitage *et al.* 2019).

### 3.5 Learning a new categorization of drugs

**Figure 4.**
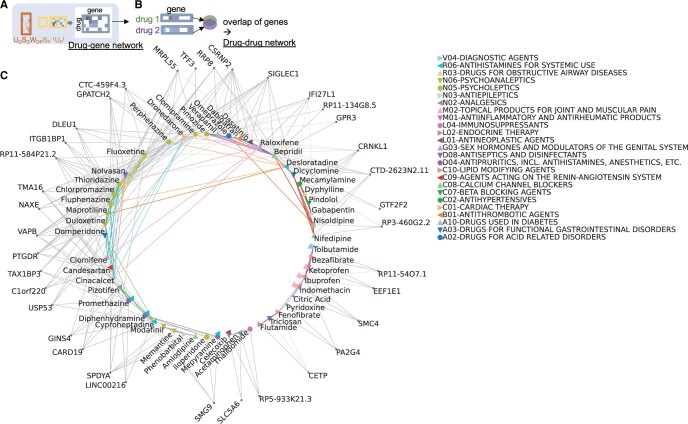
(A) Schematic of drug–gene network. (B) Schematic of creation of drug–drug network by overlap of disease genes. (C) Visualization of categorization of drugs based on their association with disease genes. Drugs are connected to their significant disease genes with gray lines (derived as in (A)). Drugs that share a significant overlap in disease genes are connected to each other with colored lines (derived as in (B)). The lines are colored based on fully connected cliques of drugs, where each color represents one unique clique. Only disease genes associated with one or more gene targets and associated with fewer than 15 drugs are shown; only drugs belonging to a fully connected clique containing at least three drugs are shown. Selected cliques discussed in the text are represented as filled polygons, i.e. verapamil, bepridil, pimozide. Drugs with available ATC categories are indicated with symbols (which are shifted slightly in order to avoid overlap).

Discovering biological effects of drugs is an open area of research. The results above point to some unexpected groupings of drugs, which we further investigate. Draphnet enables creation of a network linking drugs to their impact on disease genes (drug–disease genome matrix, Fig. 4A). Using this result, we create a novel drug–drug network: for each pair of drugs, we obtain their respective significantly associated disease genes, and then we assess whether the pair has a significant overlap of disease genes (Fig. 4B, Supplementary Table S3, section “Methods”). Then, we create a novel categorization of drugs using the network: we identify fully connected cliques of drugs, containing at least 3 drugs that are all connected with each other (Fig. 4C). This exploratory categorization is intended to be a simple application of our drug–disease genome matrix.

As expected, this categorization overlaps with known drug targets and drug effects. For example, anti-inflammatories such as ketoprofen, ibuprofen, and indomethacin cluster together, but they also connect to PPARɑ-agonists (fenofibrate, bezafibrate, Fig. 4C). PPARɑ-agonists are known to modulate inflammation (Zandbergen and Plutzky 2007). Another connected drug is pyridoxine (vitamin B-6), a nutrient with diverse effects including anti-inflammation (Ueland *et al.* 2017). In a cluster containing calcium channel blockers (bepridil, verapimil), we surprisingly find connections to pimozide, a psychiatric drug known to cause arrhythmia as a side effect. Another intriguing drug cluster connects mecamylamine, a largely discontinued antihypertensive, to pindolol, an antihypertensive beta blocker, as well as gabapentin, a central nervous system drug for seizures and nerve pain. Mecamylamine was discontinued as an antihypertensive in part because of its unintended diverse central nervous system effects, but more recently has been investigated for seizure and behavioral disorders (Nickell *et al.* 2013). We present these drug connections as a resource for further exploration.

## 4 Discussion

Our approach learns how drug molecular effects impact disease genes and result in drug effects on phenotype. We evaluate our model’s ability to predict drug effects by evaluating predictions against two data on drug side effects, SIDER and Offsides (Tatonetti *et al.* 2012). As well, we show that these predictions outperform both predictions based directly on the input data, as well as predictions of two published methods for predicting drug side effects (Oh *et al.* 2017, Galeano *et al.* 2020). Each method has its own goals: while the recommender system of Galeano *et al.* is intended to enable prediction of side effect frequencies, Draphnet is intended to explain drug effects in terms of interactions of drug and phenotype features. To evaluate if Draphnet achieves this goal, we investigated Draphnet’s learned representations of drugs: although drug targets were not used to train the model, drugs sharing targets have more similar Draphnet representations. Finally, we predicted the disease genes impacted by each drug, and showed that drugs sharing targets are enriched for drugs predicted to impact the same disease genes, showing that Draphnet’s results reflect known drug biology.

While neural networks and other supervised approaches could outperform our predictions on the same data, we focus not on maximizing predictive performance but on biological interpretability. It is worth noting that the drug–effect matrix used to train the model is, of necessity, always incomplete: we expect our putative negative training examples include some drug–phenotype relationships that have not yet been discovered. Then, accuracy may not be the best metric for evaluating the performance of the model (Galeano and Paccanaro 2018). We describe a number of predicted drug effects that are not present in SIDER, which may be false positives, but may also be true effects that have yet to be reported. On the other hand, our model does not perfectly recover all known drug effects: for instance, some drugs like pentamidine have scant effects captured by ToxCast, resulting in false negatives.

Our results show that Draphnet can provide insights into both new drug effects on phenotypes, and the biological basis of unexpected drug effects. Among the insights reported above, we suggest that neuroleptics may act by modulating *PPM1M*, a gene with growing evidence for a role in mental illness (Goes *et al.* 2015, Mallard *et al.* 2022). As well, our results point to a role of *CETP* in cholesterol-lowering therapies, also supported by recent studies (Hansen *et al.* 2010, Armitage *et al.* 2019). Many more drug–disease gene connections are compiled in Supplementary Table S1. If such testable hypotheses are supported with experimental work, these findings could suggest that other therapies impacting these genes may be effective for these health conditions. Our method is also able to suggest that drugs with disparate historic uses may in fact share common mechanisms, suggesting possibilities for drug repurposing, or explanations for drug side effects.

We have also shown the potential of two untapped data sources for drug side effect and indication discovery: ToxCast and PhenomeXcan. The PhenomeXcan resource is built on the S-PrediXcan method, which is widely cited and has been shown to replicably identify disease genes (Barbeira *et al.* 2018). While PhenomeXcan has been used to suggest possible drugs for a few diseases (So *et al.* 2017, Gerring *et al.* 2021), no previous method has integrated this information across a range of diseases to build a drug–phenotype model. Previous studies have shown that ToxCast assay results contain biologically relevant signal (Richard *et al.* 2016) and this resource has been applied for diverse research questions such as predicting effects of drugs on metabolism (Filer *et al.* 2022), reproductive disruption (Martin *et al.* 2011), or food safety (Firman *et al.* 2021). To our knowledge, ToxCast has not been used in a systematic analysis simultaneously modeling the molecular effects of a large number of drugs to discover new drug biology. Future work could also extend the method to use other data sources, such as LINCS Connectivity Map data, which comprehensively profiles the effects of compounds on gene expression in multiple cancer cell lines.

A limitation of our study is that although we aim to maximize the number of drug–phenotype pairs included, the training data size remains low considering the number of parameters we could aim to estimate. To address this issue and assess its effect on our results, we have taken steps including cross-validation and regularization; reducing the feature space to minimize the number of parameters in the model; and rigorous assessment of the resulting model, both in terms of drug–phenotype association data (including both held-out SIDER profiles and Offsides statistics), and also with independent drug target data. While regularization is expected to improve the generalizability and robustness of the model, determining the regularization settings does involve a tradeoff between false positives and false negatives (Su *et al.* 2016). There are also weaknesses in the available data. GWAS data do not yet not capture all genetic etiology contributing to disease (Tam *et al.* 2019), and there is ambiguity when assigning GWAS signals to disease genes. For instance, one gene connected to PPARγ agonists, the long noncoding RNA RP11-54O7.17 is adjacent to *PERM1*, a known effector of PPARγ (Cho *et al.* 2013). But, *PERM1* has fewer recorded eQTLs, possibly explaining its lack of signal in the PhenomeXcan data. Our approach may overlook pathogenic effects of genetics that do not relate to gene expression. Future work could re-fit the model using other comprehensive resources linking genes to disease (Watanabe *et al.* 2019). Another limitation is that ToxCast cannot capture organ level effects of compounds, but only *in vitro* readouts of likely relevance for organ and organism level biology. We expect near future resources will include analogous comprehensive screens of the effect of drugs on organoids, and our model can be easily refitted with such data. Finally, SIDER is, as mentioned above, incomplete, and biases (such as reporting bias) may influence recorded drug–phenotype relationships, impacting the model.

The results we have already provided can be a starting point for multiple new experiments and analyses. The results presented in Figshare and our Supplementary tables include: (i) predicted multiple new drug–phenotype effects, of immediate clinical interest; (ii) disease genes related to each drug, with potential for insight into drug biology and new therapeutic targets; and (iii) a novel categorization of drugs that could not only suggest drug repurposing opportunities but also point to its basis in shared pathways. Future computational work could directly analyze our results in a few ways. By projecting the drug phenome effect matrix to biological pathways, follow-up analyses can further interpret the effects of drugs. As well, similar to our work categorizing drugs, analysis of our results can show how unexpected diseases may share targetable disease networks. Another analysis could use our models to analyze possible differences between the biology of drug indications and drug side effects. In addition to reapplying our method with other data sources, as mentioned above, other analyses could build on our method. Although our implementation did not use neural networks, the design could alternatively be implemented as a simple neural network. This connection invites future advances using neural networks configurations incorporating structured gene networks (Kuenzi *et al.* 2020). Another application could adapt our model to predict personalized drug effects based on the risk allele profile of a given individual, impacting precision medicine. Both analysis of our current results, and future improvements on the method, promise to improve our understanding of the biological basis of unexpected medication effects on human health.

## Supplementary Material

vbae038_Supplementary_Data
